# Causal Association Between Gut Microbiota and Sleep Apnoea Identified by Bayesian Weighted Mendelian Randomisation

**DOI:** 10.1111/jcmm.70976

**Published:** 2026-01-07

**Authors:** Chenguang Zhang, Yicong Wang, Guanghao Yue, Bin Guo

**Affiliations:** ^1^ Graduate School of Qinghai University Xining China; ^2^ Department of Otolaryngology Qinghai University Affiliated Hospital Xining China; ^3^ Department of Gastrointestinal Oncology Qinghai University Affiliated Hospital Xining China

**Keywords:** Bayesian weighted Mendelian randomisation (BWMR), causal association, gut microbiota, OSA, probiotics, sleep apnoea, translational medicine

## Abstract

This study investigated the potential causal relationship between gut microbiota (GM) and sleep apnoea using Mendelian randomisation (MR) analysis. Summary‐level genome‐wide association (GWAS) data for 473 GM and sleep apnoea were obtained from the IEU Open GWAS database. A two‐sample MR framework was applied to assess the potential causal effects of GM on sleep apnoea. The primary analysis was conducted using the inverse variance–weighted (IVW) method, complemented by MR‐Egger regression, weighted median, weighted mode and simple mode approaches to ensure robustness. To further account for horizontal pleiotropy and weak instrument bias, Bayesian Weighted Mendelian Randomisation (BWMR) analysis was performed as a key sensitivity model. Sensitivity analyses, including heterogeneity tests and pleiotropy assessments, were conducted to evaluate the stability and reliability of the results. IVW identified 33 GM associated with sleep apnoea (*p* < 0.05); BWMR confirmed 24 with significant causal effects, including 10 showing negative (protective) and 14 showing positive (risk) associations. Sensitivity analyses supported robustness: MR‐PRESSO indicated outlier signals in 3 GM, Cochran's *Q* detected heterogeneity in 5 GM, and MR‐Egger intercept suggested directional pleiotropy in 3 GM; all remaining GM showed non‐significant sensitivity metrics. Leave‐one‐out analyses showed no single SNP disproportionately influenced the estimates, reinforcing the stability of the findings. This MR study provides genetic evidence supporting a potential causal association between GM and sleep apnoea. These findings provide new insights that may inform future research and prevention strategies.

## 1 Introduction

Obstructive sleep apnoea (OSA) is a highly prevalent sleep‐disordered breathing condition with significant public health implications. Recent epidemiological studies estimate that OSA affects nearly one billion adults worldwide [1]. Prevalence rates range widely (afflicting roughly 10%–30% of middle‐aged adults, more in high‐risk groups) and have been increasing in tandem with the global rise in obesity and ageing populations [2, 3]. OSA often remains underdiagnosed, and the disorder imposes a substantial health and economic burden, with undiagnosed OSA contributing to hundreds of billions in healthcare costs. Importantly, OSA is associated with a heightened risk of cardiovascular and metabolic comorbidities and elevated mortality; untreated OSA markedly increases the incidence of hypertension, heart disease, stroke and all‐cause mortality [4]. Key risk factors for OSA include obesity, male sex and older age [2]. Nevertheless, OSA can affect individuals across all body‐mass index categories. Emerging evidence also implicates gut microbiota (GM) dysbiosis in OSA pathophysiology, suggesting that alterations in the GM may mediate systemic inflammation and metabolic dysfunction in OSA patients [5, 6].

The GM comprises the microorganisms of the human gastrointestinal tract and is crucial for host metabolism, immunity and homeostasis [7, 8, 9, 10, 11, 12, 13]. Recent studies have revealed associations between OSA and changes in GM composition, implicating gut dysbiosis in OSA‐related comorbidities [14, 15]. Patients with OSA exhibit an altered GM, characterised by reduced microbial diversity, loss of beneficial short‐chain fatty acid‐producing bacteria, and an expansion of pro‐inflammatory taxa [16, 17]. Such dysbiosis has been linked to systemic inflammation, metabolic dysfunction and neurocognitive impairments in OSA [5, 18].

Mendelian randomisation (MR) is a genetic epidemiological approach that leverages genetic variants as instrumental variables to infer causal effects between exposures and outcomes [19, 20, 21]. By minimising confounding and reverse causation, MR provides a quasi‐experimental framework for causal inference in observational data [22, 23]. In recent years, MR has been increasingly applied to investigate microbiota–disease associations, offering novel insights beyond traditional correlation analyses [24]. The Bayesian Weighted Mendelian Randomisation (BWMR) method further enhances robustness by accounting for weak instrument bias and horizontal pleiotropy [25]. Therefore, MR provides a powerful approach to clarify the causal effects of GM alterations on OSA.

## 2 Data and Methods

### 2.1 Data

GWAS data for both the exposures and the outcome were obtained from the IEU Open GWAS database (https://opengwas.io/), an open‐access repository developed by the MRC Integrative Epidemiology Unit (IEU) at the University of Bristol, UK. The exposure dataset included the latest GWAS summary statistics of 473 GM, encompassing 10 phyla, 18 classes, 24 orders, 58 families, 143 genera, 213 species and 7 unclassified groups. The outcome dataset was retrieved from the Finnish latest R12 database (OpenGWAS ID: finn‐b‐G6_SLEEPAPNO_INCLAVO), representing sleep apnoea (FinnGen endpoint ‘G6_SLEEPAPNO_INCLAVO’, including individuals with clinical diagnoses of sleep apnoea and controls without the diagnosis). All participants in the included GWAS datasets were of European ancestry, which minimises potential population stratification bias. All GWAS summary statistics used in this study are publicly available and have received ethical approval from the respective institutional review boards of the contributing studies. Detailed information on the GWAS datasets used is presented in Table 1.

**TABLE 1:** GWAS information.

Variable	Year	Sample size	Population	Gender
Gut microbiota	2022	5959	European	Mixed gender
Sleep apnoea	2024	500,348	European	Mixed gender

### 2.2 Selection of Instrumental Variables

**FIGURE 1:**
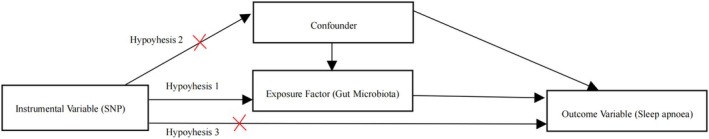
Core assumptions of Mendelian Randomisation.

Single nucleotide polymorphisms (SNPs) significantly associated with each GM were selected as instrumental variables (IVs) from the exposure GWAS at the genome‐wide significance threshold of *p* < 1 × 10^−5^ (this relaxed threshold was adopted in line with previous microbiome GWAS‐based MR studies to account for the relatively small sample size and polygenic architecture of microbiota traits, whereas all selected SNPs had *F*‐statistics > 10 to minimise weak instrument bias and ensure instrument validity). To ensure the independence of instruments, linkage disequilibrium (LD) clumping was performed with a cutoff of *r* ^2^ < 0.001 and a clumping window of 10,000 kb based on the European 1000 Genomes reference panel. Palindromic SNPs with ambiguous strand orientation were removed, and effect alleles were harmonised across exposure and outcome datasets to maintain consistency in the direction of effect. SNPs with minor allele frequency (MAF) < 0.01 were excluded to avoid potential bias caused by rare variants. The strength of each instrumental variable was assessed by calculating the *F*‐statistic, and SNPs with *F* < 10 were considered weak instruments and excluded from subsequent analyses. These stringent selection criteria ensured the validity and robustness of the IVs, satisfying the three core assumptions of MR (Figure 1): (1) the genetic variant is strongly associated with the exposure, (2) the variant is independent of confounders, and (3) the variant affects the outcome only through the exposure [26].

### 2.3 Methods

A two‐sample MR was applied to estimate the causal effects of GM on sleep apnoea. The inverse variance–weighted (IVW) method served as the primary analytical approach, as it provides the most efficient estimates when all IVs are valid. Complementary MR methods, including MR‐Egger regression, weighted median, weighted mode and simple mode, were implemented to ensure the robustness of the causal inference under different model assumptions.

GM showing statistically significant associations in the IVW analysis (*p* < 0.05) were further evaluated using the BWMR method. BWMR was used to account for horizontal pleiotropy and weak instrument bias, providing a more robust causal estimate. Associations with *p* < 0.05 in the BWMR analysis were considered statistically significant. Heterogeneity across SNPs was assessed using Cochran's *Q* statistic, and horizontal pleiotropy was examined using the MR‐Egger intercept test. The MR‐PRESSO (Mendelian Randomisation Pleiotropy RESidual Sum and Outlier) global test was also applied to detect and correct for potential outlier SNPs, while leave‐one‐out analysis was conducted to examine whether any single SNP disproportionately influenced the overall causal estimates. All statistical analyses were performed using R software (version 4.4.2) with the ‘TwoSampleMR’, ‘MRPRESSO’ and ‘BWMR’ packages.

## 3 Results

### 3.1 Mendelian Randomisation Results

**FIGURE 2:**
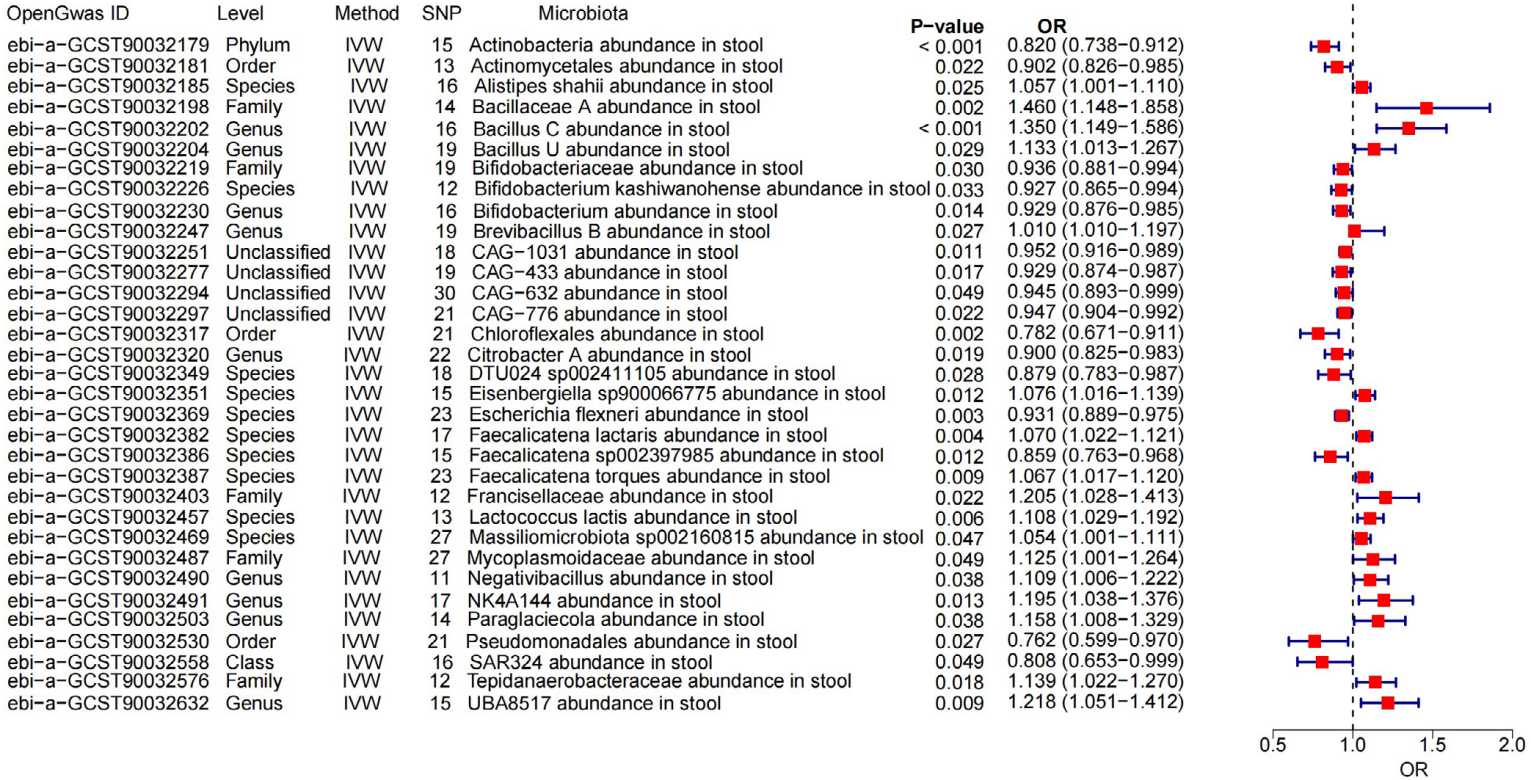
Results after the inverse variance–weighted method analysis. IVW, inverse variance–weighted.

A total of 473 GM were included in the MR analysis to explore their potential causal relationships with sleep apnoea. Based on the IVW analysis, 33 GM showed significant associations (*p* < 0.05) with sleep apnoea (Figure 2). To further verify the robustness of these findings and control for pleiotropic effects, these 33 GM were re‐evaluated using the BWMR method.

Following BWMR analysis, 24 GM remained significantly associated with sleep apnoea (*p* < 0.05). GM showing negative associations—suggesting potential protective effects—included *Actinobacteria* (*β* = −0.190, OR = 0.827, 95% CI = 0.735–0.930, *p* = 0.002), *Bifidobacterium* (*β* = −0.071, OR = 0.932, 95% CI = 0.877–0.990, *p* = 0.023), *CAG‐1031* (*β* = −0.054, OR = 0.948, 95% CI = 0.910–0.987, *p* = 0.009), *CAG‐433* (*β* = −0.073, OR = 0.930, 95% CI = 0.876–0.987, *p* = 0.017), *CAG‐776* (*β* = −0.063, OR = 0.939, 95% CI = 0.897–0.984, *p* = 0.008), *Chloroflexales* (*β* = −0.258, OR = 0.773, 95% CI = 0.657–0.909, *p* = 0.002), *DTU024 sp002411105* (*β* = −0.137, OR = 0.872, 95% CI = 0.779–0.976, *p* = 0.017), *Escherichia flexneri* (*β* = −0.083, OR = 0.921, 95% CI = 0.881–0.962, *p* < 0.001), *Faecalicatena sp002397985* (*β* = −0.161, OR = 0.851, 95% CI = 0.752–0.964, *p* = 0.011) and *SAR324* (*β* = −0.243, OR = 0.785, 95% CI = 0.625–0.985, *p* = 0.037).

Conversely, GM showing positive associations—indicating increased risk—were *Alistipes shahii* (*β* = 0.056, OR = 1.058, 95% CI = 1.013–1.104, *p* = 0.010), *Bacillaceae A* (*β* = 0.398, OR = 1.489, 95% CI = 1.145–1.937, *p* = 0.003), *Bacillus C* (*β* = 0.312, OR = 1.366, 95% CI = 1.146–1.628, *p* < 0.001), *Bacillus U* (*β* = 0.153, OR = 1.166, 95% CI = 1.038–1.309, *p* = 0.010), *Eisenbergiella sp900066775* (*β* = 0.074, OR = 1.077, 95% CI = 1.007–1.151, *p* = 0.030), *Faecalicatena lactaris* (*β* = 0.069, OR = 1.071, 95% CI = 1.024–1.122, *p* = 0.003), *Faecalicatena torques* (*β* = 0.068, OR = 1.070, 95% CI = 1.018–1.125, *p* = 0.008), *Francisellaceae* (*β* = 0.198, OR = 1.220, 95% CI = 1.046–1.422, *p* = 0.011), *Lactococcus lactis* (*β* = 0.103, OR = 1.110, 95% CI = 1.023–1.202, *p* = 0.012), *Massiliomicrobiota sp002160815* (*β* = 0.054, OR = 1.056, 95% CI = 1.002–1.112, *p* = 0.042), *NK4A144* (*β* = 0.183, OR = 1.201, 95% CI = 1.036–1.392, *p* = 0.015), *Paraglaciecola* (*β* = 0.151, OR = 1.163, 95% CI = 1.002–1.350, *p* = 0.047), *Tepidanaerobacteraceae* (*β* = 0.134, OR = 1.143, 95% CI = 1.019–1.282, *p* = 0.022) and *UBA8517* (*β* = 0.172, OR = 1.187, 95% CI = 1.023–1.378, *p* = 0.024) (Table 2).

**TABLE 2:** Results after Bayesian Weighted Mendelian Randomisation analysis.

Microbiota	Method	Beta	Or	Or_lci95	Or_uci95	*p*
*Actinobacteria*	BWMR	−0.190	0.827	0.735	0.930	0.002
*Actinomycetales*	BWMR	−0.071	0.931	0.849	1.021	0.130
*Alistipes shahii*	BWMR	0.056	1.058	1.013	1.104	0.010
*Bacillaceae A*	BWMR	0.398	1.489	1.145	1.937	0.003
*Bacillus C*	BWMR	0.312	1.366	1.146	1.628	0.000
*Bacillus U*	BWMR	0.153	1.166	1.038	1.309	0.010
*Bifidobacteriaceae*	BWMR	−0.056	0.946	0.890	1.005	0.073
*Bifidobacterium kashiwanohense*	BWMR	−0.045	0.956	0.887	1.030	0.237
*Bifidobacterium*	BWMR	−0.071	0.932	0.877	0.990	0.023
*Brevibacillus B*	BWMR	0.073	1.075	0.982	1.177	0.117
*CAG‐1031*	BWMR	−0.054	0.948	0.910	0.987	0.009
*CAG‐433*	BWMR	−0.073	0.930	0.876	0.987	0.017
*CAG‐632*	BWMR	−0.063	0.939	0.880	1.001	0.054
*CAG‐776*	BWMR	−0.063	0.939	0.897	0.984	0.008
*Chloroflexales*	BWMR	−0.258	0.773	0.657	0.909	0.002
*Citrobacter A*	BWMR	−0.088	0.916	0.836	1.003	0.059
*DTU024 sp002411105*	BWMR	−0.137	0.872	0.779	0.976	0.017
*Eisenbergiella sp900066775*	BWMR	0.074	1.077	1.007	1.151	0.030
*Escherichia flexneri*	BWMR	−0.083	0.921	0.881	0.962	0.000
*Faecalicatena lactaris*	BWMR	0.069	1.071	1.024	1.122	0.003
*Faecalicatena sp002397985*	BWMR	−0.161	0.851	0.752	0.964	0.011
*Faecalicatena torques*	BWMR	0.068	1.070	1.018	1.125	0.008
*Francisellaceae*	BWMR	0.198	1.220	1.046	1.422	0.011
*Lactococcus lactis*	BWMR	0.103	1.110	1.023	1.202	0.012
*Massiliomicrobiota sp002160815*	BWMR	0.054	1.056	1.002	1.112	0.042
*Mycoplasmoidaceae*	BWMR	0.117	1.124	0.995	1.271	0.061
*Negativibacillus*	BWMR	0.085	1.089	0.992	1.194	0.073
*NK4A144*	BWMR	0.183	1.201	1.036	1.392	0.015
*Paraglaciecola*	BWMR	0.151	1.163	1.002	1.350	0.047
*Pseudomonadales*	BWMR	−0.234	0.791	0.616	1.016	0.066
*SAR324*	BWMR	−0.243	0.785	0.625	0.985	0.037
*Tepidanaerobacteraceae*	BWMR	0.134	1.143	1.019	1.282	0.022
*UBA8517*	BWMR	0.172	1.187	1.023	1.378	0.024

*Note:* BWMR, Bayesian Weighted Mendelian Randomisation, for interpretability, *β* coefficients from BWMR were exponentiated to obtain odds ratios (OR = e^*β*^), indicating the change in sleep apnoea risk per genetically predicted standard deviation (SD) increase in microbial abundance. Red‐colored values indicate statistically significant results (*p* < 0.05).

**FIGURE 3:**
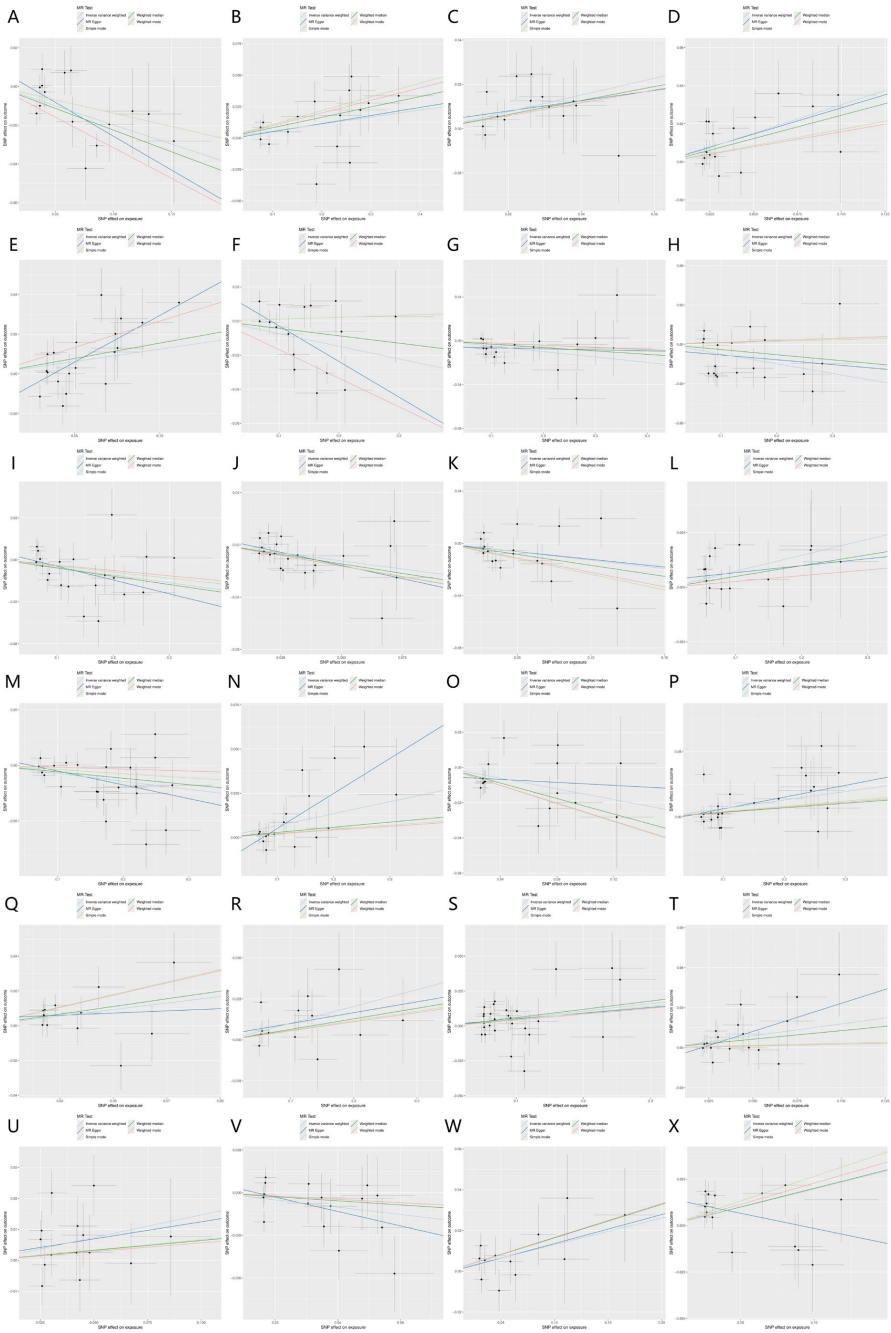
(A–X) Scatter plot of the causal association of 24GM on sleep apnoea. (A) Actinobacteria; (B) *Alistipes shahii* ; (C) Bacillaceae A; (D) Bacillus C; (E) Bacillus U; (F) Bifidobacterium; (G) CAG‐1031; (H) CAG‐433; (I) CAG‐776; (J) Chloroflexales; (K) DTU024 sp002411105; (L) Eisenbergiella sp900066775; (M) Escherichia flexneri (N) Faecalicatena lactaris (O) Faecalicatena sp002397985; (P) Faecalicatena torques; (Q) Francisellaceae; (R) *Lactococcus lactis* ; (S) Massiliomicrobiota sp002160815 (T) NK4A144; (U) Paraglaciecola; (V) SAR324; (W) Tepidanaerobacteraceae; (X) UBA8517.

**FIGURE 4:**
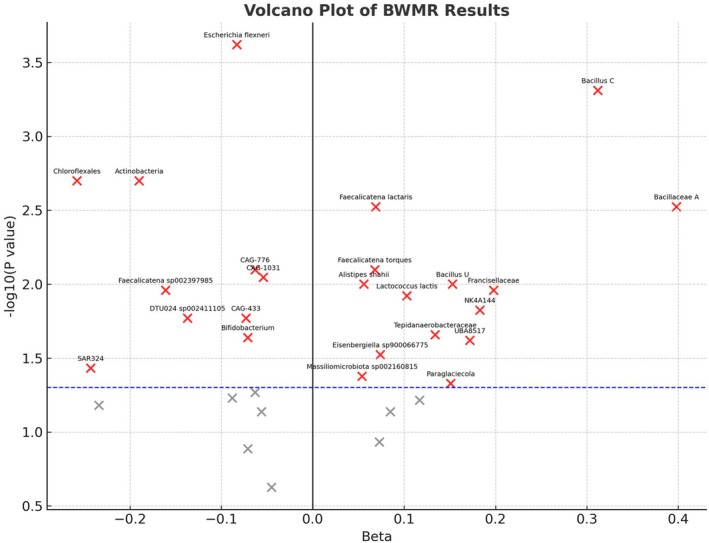
Volcano plot of BWMR results. BWMR, Bayesian Weighted Mendelian Randomisation.

All GM with statistically significant associations in the BWMR analysis (*p* < 0.05) were further visualised using scatter plots and volcano plots (Figures 3 and 4). These plots illustrate both the direction and magnitude of causal effects, providing an overview of GM with potential protective and risk effects on sleep apnoea.

### 3.2 Sensitivity Analysis

**FIGURE 5:**
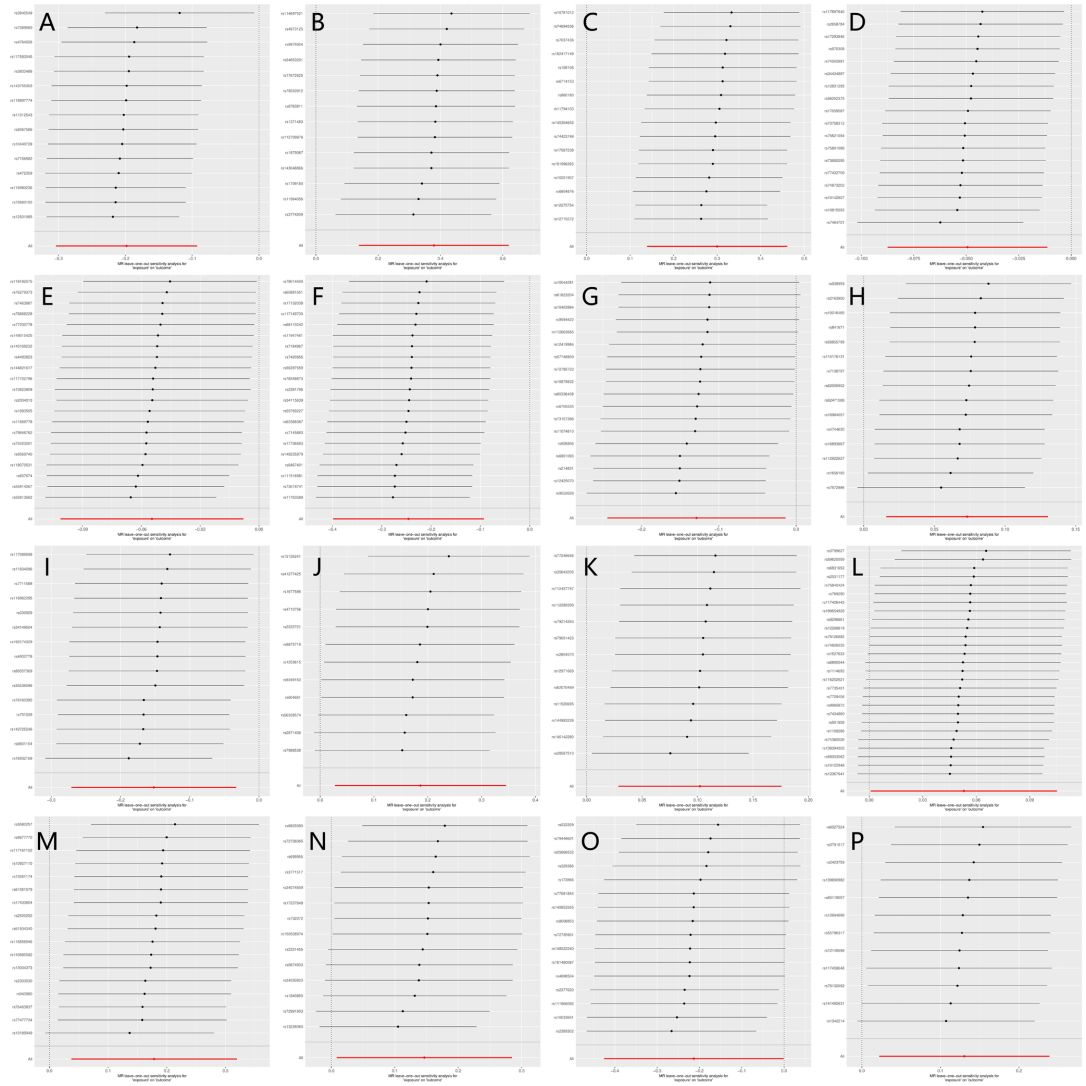
(A–P) Leave‐one‐out plot of the causal association of 16GM on sleep apnoea. (A) Actinobacteria; (B) Bacillaceae A; (C) Bacillus C; (D) CAG‐1031; (E) CAG‐776; (F) Chloroflexales; (G) DTU024 sp002411105; (H) Eisenbergiella sp900066775; (I) Faecalicatena sp002397985; (J) Francisellaceae; (K) *Lactococcus lactis* ; (L) Massiliomicrobiota sp002160815; (M) NK4A144 (N) Paraglaciecola (O) SAR324; (P) Tepidanaerobacteraceae.

After screening by both the IVW and BWMR methods, 24 GM were subjected to sensitivity analyses. We used the MR‐PRESSO global test to detect outlier SNPs, Cochran's *Q* (for MR‐Egger and IVW) to assess heterogeneity, and the MR‐Egger intercept to test directional pleiotropy. As summarised in Table 3, most GM showed no evidence of heterogeneity (*Q*_*p* ≥ 0.05) or directional pleiotropy (Egger intercept *p* ≥ 0.05), suggesting that the causal estimates were generally robust. However, several GM exhibited signals that warranted caution. Outlier effects were observed for *Bifidobacterium* (*p* = 0.003), *CAG‐433* (*p* = 0.001) and *Escherichia flexneri* (*p* = 0.027). Evidence of heterogeneity was detected in *Alistipes shahii* (MR‐Egger *Q p* = 0.049), *Bifidobacterium* (MR‐Egger *Q p* = 0.018; IVW *Q p* = 0.002), *CAG‐433* (MR‐Egger/IVW *Q p* = 0.001/0.001), *Escherichia flexneri* (MR‐Egger/IVW *Q p* = 0.023/0.020) and *Faecalicatena torques* (MR‐Egger/IVW *Q p* = 0.033/0.041). In addition, directional pleiotropy was indicated for *Bacillus U* (intercept −0.018, *p* = 0.019), *Faecalicatena lactaris* (intercept −0.015, *p* = 0.027) and *UBA8517* (intercept 0.015, *p* = 0.011). For the remaining GM, all sensitivity metrics were non‐significant. The leave‐one‐out analysis (Figure 5) further confirmed that no single SNP had a disproportionate influence on the causal estimates, supporting the reliability and robustness of the observed causal associations between GM and sleep apnoea.

**TABLE 3:** Sensitivity analysis results.

Microbiota	MR‐PRESSO global test *p*	Heterogeneity test		Horizontal pleiotropy test	
		MR Egger *Q*_*p*	Inverse variance weighted *Q*_*p*	Egger_intercept	Egger_intercept, *p*
*Actinobacteria*	0.088	0.112	0.082	0.009	0.219
*Alistipes shahii*	0.079	0.049	0.069	−0.001	0.851
*Bacillaceae A*	0.611	0.535	0.564	0.003	0.633
*Bacillus C*	0.234	0.120	0.159	0.001	0.900
*Bacillus U*	0.085	0.278	0.065	−0.018	0.019
*Bifidobacterium*	0.003	0.018	0.002	0.018	0.060
*CAG‐1031*	0.506	0.505	0.510	−0.005	0.351
*CAG‐433*	0.001	0.001	0.001	−0.004	0.614
*CAG‐776*	0.123	0.105	0.105	0.005	0.369
*Chloroflexales*	0.477	0.440	0.454	0.004	0.384
*DTU024 sp002411105*	0.249	0.185	0.233	−0.000	0.937
*Eisenbergiella sp900066775*	0.435	0.381	0.424	0.003	0.531
*Escherichia flexneri*	0.027	0.023	0.020	0.007	0.314
*Faecalicatena lactaris*	0.286	0.603	0.267	−0.015	0.027
*Faecalicatena sp002397985*	0.432	0.406	0.412	−0.005	0.354
*Faecalicatena torques*	0.058	0.033	0.041	−0.003	0.649
*Francisellaceae*	0.279	0.221	0.268	0.004	0.617
*Lactococcus lactis*	0.350	0.249	0.298	0.003	0.615
*Massiliomicrobiota sp002160815*	0.089	0.068	0.086	0.001	0.853
*NK4A144*	0.432	0.479	0.419	−0.009	0.192
*Paraglaciecola*	0.213	0.138	0.183	0.001	0.868
*SAR324*	0.186	0.193	0.194	0.006	0.367
*Tepidanaerobacteraceae*	0.735	0.628	0.711	−0.001	0.911
*UBA8517*	0.063	0.381	0.055	0.015	0.011

*Note:* Red‐colored values indicate statistically significant results (*p* < 0.05).

## 4 Discussion

MR analysis provides novel evidence supporting a causal link between the GM and OSA. We identified multiple GM whose genetically predicted abundance influences OSA risk. Notably, higher levels of *Actinobacteria* (a phylum that includes *Bifidobacterium*) were associated with a lower likelihood of OSA, whereas increases in certain *Firmicutes* (e.g., *Bacillus* genus from the *Bacillaceae* family) corresponded to a higher OSA risk. These findings align with and extend prior observational studies that reported gut dysbiosis in OSA patients [27, 28]. For example, severe OSA has been linked to diminished short‐chain fatty acid (SCFA)‐producing bacteria (like *Faecalibacterium* and *Bacteroides* spp.) alongside an increased *Firmicutes*‐to‐*Bacteroidetes* ratio [17]. A previous MR study by Liu et al. included 211 GM, whereas our analysis encompassed 473 taxa, offering a broader and more detailed assessment that consistently identified *Bifidobacterium* as a protective factor against OSA. In addition, the sensitivity analyses revealed that a few taxa—such as *Bifidobacterium*, *CAG‐433*, *Escherichia flexneri*, *Bacillus U*, *Faecalicatena lactaris* and *UBA8517*—showed moderate heterogeneity or potential horizontal pleiotropy. These findings suggest that their causal interpretations should be treated cautiously. The variability may reflect the complex and multifactorial nature of host–microbe interactions, where some bacterial groups influence both metabolic and inflammatory pathways related to OSA [6]. Although BWMR down‐weighted weak or pleiotropic instruments, residual biological overlap cannot be completely excluded. Nevertheless, the direction and magnitude of effects were largely consistent across IVW, MR‐Egger and BWMR models, supporting the robustness of the overall causal pattern between GM and OSA.

Several mechanisms likely mediate how GM perturbations influence OSA risk and severity. First, metabolite‐driven modulation is central: beneficial microbes such as *Bifidobacterium* and butyrate producers ferment dietary fibres to short‐chain fatty acids (SCFAs). SCFAs, particularly butyrate, reinforce the gut barrier, dampen systemic inflammation, and improve metabolic homeostasis—all of which counteract obesity and inflammation, the key contributors to OSA [6, 29, 30, 31, 32, 33, 34]. In contrast, a dysbiotic microbiome enriched in Gram‐negative bacteria can release lipopolysaccharide (LPS) into circulation when intestinal permeability is impaired, activating the TLR4/NF‐κB pathway and promoting systemic inflammation and endothelial dysfunction [35]. Indeed, experimental studies have shown that transplanting OSA‐associated microbiota increases plasma LPS and vascular inflammation in mice, resulting in hypertension. Clinically, OSA patients exhibit elevated gut permeability biomarkers (e.g., D‐lactate, I‐FABP) correlating with the abundance of pro‐inflammatory taxa and the depletion of beneficial SCFA‐producing bacteria [36]. Furthermore, the GM can influence metabolic and neurohumoral regulation: loss of branched‐chain amino acid (BCAA)‐catabolising bacteria may elevate plasma BCAA levels, contributing to insulin resistance, while microbial bile acids modulate circadian receptors and respiratory control, linking microbiota to host sleep regulation [37]. In sum, multiple converging pathways—metabolic, barrier, inflammatory and neurohumoral—may channel gut microbial dysbiosis into increased OSA susceptibility.

Our findings carry several important clinical implications. First, they suggest that the gut microbiota could be targeted as a novel therapeutic avenue in OSA management. Conventional OSA treatments (such as continuous positive airway pressure, mandibular devices, or surgery) primarily address the anatomical and neural aspects of airway obstruction, but do not tackle the systemic metabolic inflammation that often accompanies OSA. If specific bacterial taxa indeed causally influence OSA risk, modifying the gut microbiome offers a complementary strategy to alleviate OSA and its cardiometabolic complications [28, 38]. Microbiota‐targeted interventions—including probiotics, prebiotic dietary fibres, synbiotics or even faecal microbiota transplantation—hold promise for improving OSA outcomes. For example, enrichment of Actinobacteria like Bifidobacterium (through probiotic supplementation or high‐fibre diets) might help with weight control and reduce airway inflammatory edema, thereby lowering OSA severity [39, 40]. There is growing interest in such strategies; recent reviews highlight that manipulating the gut flora could attenuate OSA‐related hypertension and oxidative stress by restoring a healthy gut metabolite profile [28, 41]. Second, the GM and its metabolites may serve as potential biomarkers for OSA risk stratification and treatment response. Specific GM, such as SCFA‐producing bacteria or *Bifidobacteriaceae*, could indicate increased susceptibility to OSA or more severe disease phenotypes. This highlights the possibility of developing non‐invasive stool‐based microbial signatures to support OSA diagnosis and personalised therapy. Integrating conventional OSA treatments with targeted dietary or probiotic modulation tailored to an individual's microbiome may enhance outcomes and reduce cardiometabolic comorbidities [42, 43]. While no microbiome‐focused therapy is yet part of standard OSA care, our findings lay the groundwork for such innovations. In summary, recognising the gut microbiota as an integral player in OSA opens the door to preventive and therapeutic strategies that go beyond the airway, targeting systemic health to combat this multifactorial disease. This aligns with a more holistic view of OSA, treating it not just as a localised airway disorder but as a condition interconnected with metabolism and immunity. Finally, our study underscores the importance of multidisciplinary management—for instance, close collaboration between sleep specialists, dietitians and microbiome experts—to translate these insights into patient care.

The study has several limitations. First, the GWAS data used in this MR analysis were primarily derived from individuals of European ancestry, which may limit the generalizability of our findings to other populations with different genetic backgrounds and microbiome compositions. Second, the genetic instruments capture only part of the variation in microbial taxa, and some unclassified groups (e.g., CAG taxa) have poorly defined functions, preventing precise mechanistic interpretation. Third, OSA was treated as a binary outcome; thus, we could not assess the effects of GM on disease severity or phenotypic heterogeneity. Finally, while MR strengthens causal inference, it cannot replace randomised clinical trials. Further intervention studies—such as dietary or probiotic trials—are warranted to confirm whether modulating the GM can improve OSA outcomes. Despite these limitations, this study provides genetic evidence linking GM to OSA risk, offering a foundation for future translational research.

## 5 Conclusion

In conclusion, this MR study explored the potential causal association between GM and sleep apnoea using large‐scale genetic data. The results provide supportive evidence for a link between gut microbial composition and the risk of sleep apnoea at the population level. These findings contribute to a better understanding of the microbiome's role in sleep‐related disorders. Further validation in diverse cohorts and prospective clinical studies is warranted to strengthen these observations and guide future translational research.

## Author Contributions

**Bin Guo:** writing – review and editing, visualization, validation, funding acquisition, supervision. **Guanghao Yue:** visualization, validation, funding acquisition, supervision, writing – review and editing. **Chenguang Zhang:** conceptualization, investigation, methodology, software, formal analysis, project administration, resources, data curation, writing – original draft. **Yicong Wang:** conceptualization, writing – original draft.

## Funding

The study was supported by the 2021 Qinghai Kunlun Elite High‐end Innovation and Entrepreneurship Talent Program (Grant n° 2021‐13), and the 2025 Kunlun Talents · High level Health Talents Project.

## Conflicts of Interest

The authors declare no conflicts of interest.

## Data Availability

The data that support the findings of this study are available in IEUOpenGWAS at https://gwas.mrcieu.ac.uk, reference number finn‐b‐G6_SLEEPAPNO_INCLAVO. These data were derived from the following resources available in the public domain: ‐finn‐b‐G6_SLEEPAPNO_INCLAVO, https://gwas.mrcieu.ac.uk.
